# A common SNP in the *UNG* gene decreases ovarian cancer risk in *BRCA2* mutation carriers

**DOI:** 10.1002/1878-0261.12470

**Published:** 2019-03-01

**Authors:** Juan Miguel Baquero, Carlos Benítez‐Buelga, Victoria Fernández, Miguel Urioste, Jose Luis García‐Giménez, Rosario Perona, the CIMBA Consortium, Javier Benítez, Ana Osorio

**Affiliations:** ^1^ Human Genetics Group Spanish National Cancer Research Centre (CNIO) Madrid Spain; ^2^ Helleday Laboratory Department of Oncology‐Pathology Karolinska Institutet Solna Sweden; ^3^ Spanish Network on Rare Diseases (CIBERER) Madrid Spain; ^4^ Familial Cancer Clinical Unit Spanish National Cancer Research Centre (CNIO) Madrid Spain; ^5^ Department of Physiology Faculty of Medicine and Dentistry Universitat de Valencia, Mixed Unit CIPF‐INCLIVA Spain; ^6^ Biomedical Research Institute Alberto Sols (CSIC‐UAM) Madrid Spain; ^7^ Consortium of Investigators of Modifiers of BRCA1/2; ^8^ Genotyping Unit (CEGEN) Spanish National Cancer Research Centre (CNIO) Madrid Spain

**Keywords:** *BRCA2*, cancer risk modifier, DNA damage, oxidative stress susceptibility, telomere damage, uracil‐DNA glycosylase

## Abstract

Single nucleotide polymorphisms (SNPs) in DNA glycosylase genes involved in the base excision repair (BER) pathway can modify breast and ovarian cancer risk in *BRCA1* and *BRCA2* mutation carriers. We previously found that SNP rs34259 in the uracil‐DNA glycosylase gene (*UNG*) might decrease ovarian cancer risk in *BRCA2* mutation carriers. In the present study, we validated this finding in a larger series of familial breast and ovarian cancer patients to gain insights into how this *UNG* variant exerts its protective effect. We found that rs34259 is associated with significant *UNG* downregulation and with lower levels of DNA damage at telomeres. In addition, we found that this SNP is associated with significantly lower oxidative stress susceptibility and lower uracil accumulation at telomeres in *BRCA2* mutation carriers. Our findings help to explain the association of this variant with a lower cancer risk in *BRCA2* mutation carriers and highlight the importance of genetic changes in BER pathway genes as modifiers of cancer susceptibility for *BRCA1* and *BRCA2* mutation carriers.

**Abbreviations**
AP: apurinic/apyrimidinic
BER: base excision repair
DSB: double‐strand break
dTTP: deoxythymidine triphosphate
dUTP: deoxyuridine triphosphate
FBOC: familial breast and ovarian cancer
FPG: formamidopyrimidine‐DNA glycosylase
HR: hazard ratio
LCLs: lymphoblastoid cell lines
PARP: poly(ADP‐ribose) polymerase
SNP: single nucleotide polymorphism
TL: telomere length
UNG: uracil‐DNA glycosylase

## 1 Introduction

Women carrying germline mutations in the *BRCA1* and *BRCA2* genes have a high lifetime risk of developing breast, ovarian, and other cancers (Milne *et al*., 2008). However, mutation carriers show considerable differences in disease manifestation, and this suggests the existence of other genetic or environmental factors that modify the risk of cancer development. BRCA proteins are involved in double‐strand break (DSB) DNA repair through the homologous recombination pathway (O'Donovan and Livingston, 2010), and cells harboring mutations in these genes are dependent on other DNA repair mechanisms. In this regard, we have shown that single nucleotide polymorphisms (SNPs) in genes from the base excision repair (BER) pathway can modify breast or ovarian cancer susceptibility in *BRCA1* and *BRCA2* mutation carriers (Osorio *et al*., 2014).

The BER pathway corrects base lesions that result from deamination, oxidation, or methylation (Xue *et al*., 2016). BER is initiated by DNA glycosylases that cleave the *N*‐glycosylic bond between the sugar and the base, and release the damaged base to form an abasic site, also termed an apurinic/apyrimidinic (AP) site (Maynard *et al*., 2009). A deficiency in BER can give rise to an accumulation of DSBs, which in the presence of a defective *BRCA1* or *BRCA2* background can persist and lead to cell cycle arrest or cell death. A synthetic lethal interaction was described between the *BRCA1/2* genes and poly(ADP‐ribose) polymerase (*PARP1*) involved in the BER pathway, with BRCA‐deficient cells being extremely sensitive to PARP1 inhibitors (Bryant *et al*., 2005; Farmer *et al*., 2005).

On the other hand, the BER pathway is essential for maintaining telomere integrity in mammals (Jia *et al*., 2015). Telomeres are susceptible to uracil misincorporation, which is primarily recognized and removed by the uracil‐DNA glycosylase (UNG) (Cortizas *et al*., 2016). Due to the presence of long arrays of TTAGGG repeats, uracil can appear in telomeric DNA by misincorporation of deoxyuridine triphosphate (dUTP) instead of deoxythymidine triphosphate (dTTP) opposite adenine or by deamination of cytosine to uracil opposite guanine (Krokan *et al*., 2002). Accumulation of uracil interferes with telomere homeostasis, and UNG‐initiated BER is necessary for the preservation of telomere integrity (Vallabhaneni *et al*., 2015).

In view of the above, we hypothesized that SNPs in DNA glycosylase genes might interfere with telomere maintenance and thus contribute to the risk of developing cancer. Supporting this idea, we reported that variant rs2304277, located in the 3′‐UTR of the glycosylase gene *OGG1*, is associated with higher ovarian cancer risk in *BRCA1* mutation carriers, probably due to transcriptional downregulation of *OGG1* and increased DNA damage and telomere instability (Benítez‐Buelga *et al*., 2016). Similarly, we analyzed variant rs804271, previously associated with increased breast cancer risk in *BRCA2* mutation carriers (Osorio *et al*., 2014), which is located within the promoter region of the glycosylase gene *NEIL2*. The modifier effect of this variant may be due to its negative impact on the performance of the NEIL2 enzyme, leading to an accumulation of oxidative lesions at telomeres (Benítez‐Buelga *et al*., 2017).

In the present study, we aimed to explain the molecular basis of the protective effect exerted by a SNP located in the 3′‐UTR of the *UNG* gene (rs34259) in *BRCA2* mutation carriers (Osorio *et al*., 2014). For that purpose, we explored the effects of the SNP on UNG activity and expression levels, and its possible involvement in telomere integrity.

## 2 Materials and methods

### 2.1 Patients and healthy controls

The study was performed in accordance with the principles of the Declaration of Helsinki. All patients and controls signed an appropriate informed consent form, and the proposal was approved by the ethics committee at the Fuenlabrada University Hospital, Madrid, Spain.

We studied a familial breast and ovarian cancer (FBOC) series of 344 individuals from 173 families meeting high‐risk criteria, and screened for deleterious mutations in the *BRCA1* and *BRCA2* genes, as reported previously (Milne *et al*., 2008). Thirty‐two families carried a deleterious mutation in *BRCA1*, 31 in *BRCA2*, and 110 did not carry any mutation in either of these two genes (BRCAX families). One hundred eleven controls were included who were relatives of *BRCA1/2* mutation carriers, did not have personal cancer antecedents, and did not harbor the corresponding familial mutation in the *BRCA1* or *BRCA2* genes. The different traits studied in this series are detailed in Table 1.

**Table 1:** Characteristics of the FBOC series and the number of persons studied for the indicated traits

	BRCA1	BRCA2	BRCAX^a^	Controls^b^	Total (FBOC)
Families	32	31	110	–	173
Healthy carriers	25	34	–	–	59
Cancer cases	26	28	120	–	174
SNP rs34259 genotyping	51	63	120	110	344
*UNG* mRNA expression	37	53	104	83	277
UNG protein expression	–	20	–	10	30
Uracil at telomeres	42	63	115	108	328
Telomere oxidation	23	19	68	62	172
Protein carbonylation	29	27	31	20	107
Telomere length	36	32	85	61	214
Telomerase activity	13	15	–	47	75

^a^ Non‐BRCA1/2 families. ^b^ Controls were relatives without cancer antecedents and negative for BRCA1/2 mutations.

### 2.2 DNA extraction and genotyping of SNP rs34259

DNA was extracted from peripheral blood of FBOC patients using the Maxwell^®^ FSC Instrument (Promega, Madison, WI, USA) following the manufacturer's instructions and quantified by the PicoGreen^®^ fluorometric assay (Thermo Fisher Scientific, Waltham, MA, USA).

Single nucleotide polymorphism genotyping was carried out using a KASPar probe specifically designed for rs34259 (LGC Genomics, Berlin, Germany). Allelic discrimination assays were performed in duplicate using the 7900HT Fast Real‐Time PCR System (Applied Biosystems, Foster City, CA, USA) and the Abi QuantStudio 6 Flex Real‐Time PCR System (Applied Biosystems) following the instrument‐specific conditions detailed by the manufacturer (LGC Genomics).

### 2.3 RNA expression analysis

RNA was extracted from peripheral blood mononuclear cells using TRIzol^®^ Reagent (Thermo Fisher Scientific). RNA quantity and quality were assessed by NanoDrop^®^ (ND‐1000 V3.7.1; Thermo Fisher Scientific). The High‐Capacity cDNA Reverse Transcription Kit (Applied Biosystems) was utilized for cDNA synthesis following the manufacturer's instructions.

The human *UNG* gene encodes both nuclear (UNG2) and mitochondrial (UNG1) forms of uracil‐DNA glycosylase (Nilsen *et al*., 1997). We designed specific primers to quantify total *UNG* mRNA expression and the relative expression of each isoform. Two microliters of cDNA at a final concentration of 10 ng·μL^−1^ was mixed with GoTaq^®^ qPCR MasterMix 1× (Promega) and 1 μm cDNA primers of each pair of primers (F/R) in a final volume reaction of 10 μL. Primers used are listed in Table S1. The amplification conditions consisted of an initial step at 95 °C for 10 min, followed by 40 cycles of 10 s at 95 °C and 1 min at 65 °C. Each qPCR was performed in triplicate including no‐template controls in an Abi QuantStudio 6 Flex Real‐Time PCR System (Applied Biosystems). Relative *UNG/UNG1/UNG2* mRNA expression was calculated using the $2^{\Delta \Delta C_{t}}$ method for qPCR analysis after normalization with the housekeeping gene *GAPDH* using the quantstudio™ Real‐Time PCR Software (Applied Biosystems).

### 2.4 Western blotting

The expression of UNG1 was quantified by western blot analysis in a subset of controls (*n* = 10) and *BRCA2* mutation carriers (*n* = 20) from the FBOC series. Briefly, peripheral blood mononuclear cells were isolated from whole blood using TRIzol^®^ Reagent (Thermo Fisher Scientific) according to manufacturer's instructions. Cell lysates were prepared in RIPA buffer (Sigma‐Aldrich, San Luis, MO, USA) in the presence of a protease inhibitor cocktail (Roche, Basel, Switzerland). Total protein concentration was determined using the Pierce BCA Protein Assay Kit (Thermo Fisher Scientific) following the manufacturer's instructions. Sixty micrograms of protein was analyzed by SDS/PAGE and transferred to Immobilon‐FL membranes (Millipore, Burlington, MA, USA). Membranes were blocked in TBS‐T (50 mm Tris/HCl, 150 mm NaCl, pH 7.5 plus 0.2% Tween‐20) and 5% nonfat milk for 1 h at RT. Blots were probed with the following primary antibodies: mouse anti‐UNG (#TA503563; OriGene, Rockville, MD, USA) at 1/1000 dilution and mouse anti‐actin (A2228; Sigma‐Aldrich) at 1/10 000 dilution in TBS‐T containing 5% nonfat milk. Anti‐mouse IgG‐HRP (Dako, Glostrup, Denmark) was used as the secondary antibody, and the immunoblots were developed using Immobilon Classico Western HRP substrate (Millipore). Each western blot was performed in quadruplicate. Images were analyzed using imagej software (NIH Image, Bethesda, MD, USA), and UNG1 protein level was normalized by actin.

In parallel, given that UNG2 protein levels in peripheral blood mononuclear cells from the FBOC series were too low to analyze their relative expression, we also performed western blot analyses of a previously described set of 18 lymphoblastoid cell lines (LCLs) (Vaclová *et al*., 2015) proceeding from *BRCA1* mutation carriers and controls following the same protocol.

### 2.5 Measurement of telomere damage

#### 2.5.1 Oxidative DNA damage within telomeres

We used a qPCR‐based method previously described to evaluate the accumulation of oxidative lesions within telomeric DNA based on differences in PCR kinetics between template DNA digested by formamidopyrimidine‐DNA glycosylase (FPG) and undigested DNA (O'Callaghan *et al*., 2011). Incubation and qPCR amplification of genomic DNA were performed as described by O'Callaghan *et al*. (2011) to estimate oxidative DNA damage levels at telomeres and the 36B4 locus.

#### 2.5.2 Quantification of uracil accumulation at telomeres

The telomere oxidation protocol (O'Callaghan *et al*., 2011) can be adapted to quantify the accumulation of different base lesions by incubating the DNA with other glycosylases that are sensitive to other specific base lesions. We used UNG to measure the accumulation of uracil, which is recognized and excised by this enzyme (Hegde *et al*., 2008), at telomeres.

Due to the high affinity of UNG for DNA (Zharkov *et al*., 2010), we optimized the protocol using a low UNG concentration and decreasing DNA amounts and incubation times. One hundred and eighty nanograms of genomic DNA was incubated in the absence or presence of 130 nm UNG (provided by T. Helleday, Karolinska Institutet, Stockholm, Sweden) in reaction buffer (25 mm Tris/HCl pH 8.0, 15 mm NaCl, 2 mm MgCl_2_, and 0.0025% Tween‐20) for 30 min at 37 °C. The reaction was stopped by incubation at 95 °C for 5 min. qPCR analysis was performed on 10 ng of digested or undigested genomic DNA using the same reagents and conditions as described in the original protocol for FPG (O'Callaghan *et al*., 2011).

### 2.6 Immunodetection of oxidized proteins

Oxidized proteins in plasma samples were detected by measuring the levels of carbonylated proteins as previously described (García‐Giménez *et al*., 2012). Carbonylated proteins are a widely used biomarker of chronic oxidative stress (Fedorova *et al*., 2013).

### 2.7 Telomere length measurement

Telomere length (TL) was quantified by high‐throughput quantitative fluorescence *in situ* hybridization (HT‐QFISH) with automated fluorescence microscopy as previously described (Canela *et al*., 2007). Because TL is strongly heritable (Pooley *et al*., 2013), BRCA status, the presence or absence of the SNP, and TL were assessed in the same member of each family. Whenever possible, we used the index case, and if this sample was not available, we used the most recently genotyped individual. As we previously demonstrated that chemotherapy affects TL (Benítez‐Buelga *et al*., 2015), we excluded patients from the analysis who were undergoing this treatment.

### 2.8 Telomerase assay

Protein extracts were obtained from peripheral blood mononuclear cells cultured in RPMI supplemented with 20% fetal bovine serum and phytohemagglutinin during 4–5 days, according to the recommendations of the manufacturer of the TRAPeze telomerase detection kit (Millipore). The average telomerase activity was determined in each sample using 0.5, 0.25, and 0.125 μg of protein extract and normalized with the internal control included in the assay. Because telomerase activity can be affected by chemotherapeutic agents (Benítez‐Buelga *et al*., 2015), we excluded all patients who received chemotherapy at any time during their lifetime.

### 2.9 Statistical analysis

To evaluate the effect of the SNP for each of the studied variables, we considered heterozygotes and homozygotes (GC/CC) as a single group, as the cancer modifier effect of rs34259 acts in a dominant fashion in *BRCA2* mutation carriers (Osorio *et al*., 2014).

Pearson's chi‐squared test was used to calculate whether the frequency of the SNP among the FBOC groups was significantly different from the frequency reported in the 1000 Genomes Project for the Iberian subpopulation (Zerbino *et al*., 2018). The Spearman correlation test was used to establish whether correlations between variables were statistically significant. We performed linear regression analysis to test whether cancer antecedents in *BRCA1* and *BRCA2* mutation carriers were associated with any of the variables evaluated in this study, but we did not find significant differences (*P* < 0.05) between healthy *BRCA1* and *BRCA2* carriers or cancer cases. Hence, we did not stratify for cancer status in these groups (Table S2).

The Kolmogorov–Smirnov test was used to evaluate whether the data sets were normally distributed. For comparative analyses, statistically significant differences were assessed by an unpaired *t*‐test for normal distributions and the Mann–Whitney *U*‐test for non‐normal distributions. Linear regression analysis including the *UNG* SNP as explanatory variable was run to test whether this SNP affected the variables studied. The effect size of the studied variant was defined as the slope of the linear regression line and was computed as the effect of the alternative allele (C) relative to the reference allele (G).

Statistical calculations and graphs were done using the spss software package version 19.0 (IBM, Armonk, NY, USA) and graphpad prism 5.03 (GraphPad Software Inc, San Diego, CA, USA). In all analyses, a 2‐tailed *P* value < 0.05 was considered statistically significant.

## 3 Results

### 3.1 Association study, validation, and fine mapping

In a previous study, using a tagging SNP approach in a large series of *BRCA1* and *BRCA2* mutation carriers (*n* = 23 463) from the CIMBA consortium, we found that SNP rs34259 showed the strongest association with ovarian cancer risk among all SNPs covering the *UNG* gene (tagged or imputed): HR: 0.80, 95% CI: 0.69–0.94, *P* = 7.6 × 10^−3^ (Osorio *et al*., 2014). This association was confirmed in a larger series of *BRCA2* mutation carriers (4291 new cases) from the OncoArray Consortium (Amos *et al*., 2017) (HR: 0.84, *P* = 7.6 × 10^−3^).

SNP rs34259 is located in the 3′UTR of the *UNG* gene, 2.4 kb downstream of the translation termination codon. Using haploreg v4.1 (Ward and Kellis, 2012), we were not able to detect a more plausible causal SNP among those in high linkage disequilibrium with rs34259 according to their predicted regulatory features (Table S3). Indeed, rs34259 has been identified as a *trans* expression quantitative trait locus (eQTL) SNP that decreased *UNG* gene expression in two independent eQTL studies (Ardlie *et al*., 2015; Westra *et al*., 2013) and we considered it the best candidate.

We genotyped SNP rs34259 in the FBOC sample set to evaluate its association with the studied variables. Genotype distributions were in Hardy–Weinberg equilibrium (χ^2^ = 0.03; *P* = 0.86). The different groups of cases and controls presented similar genotype and allele frequencies, not statistically different from the frequencies reported in the *1000 Genomes Project* for the Iberian subpopulation (Zerbino *et al*., 2018) (Table S4).

### 3.2 rs34259 is associated with lower *UNG* mRNA and protein levels

We first analyzed the SNP effect on transcriptional regulation in different tissues using the GTEx eQTL web server (Carithers *et al*., 2015). We found significantly decreased *UNG* mRNA levels associated with SNP rs34259 in several tissues, including breast (effect size = −0.17; *P* = 0.023) and blood (effect size = −0.19; *P* < 0.0001; Table S5).

**Figure 1:**
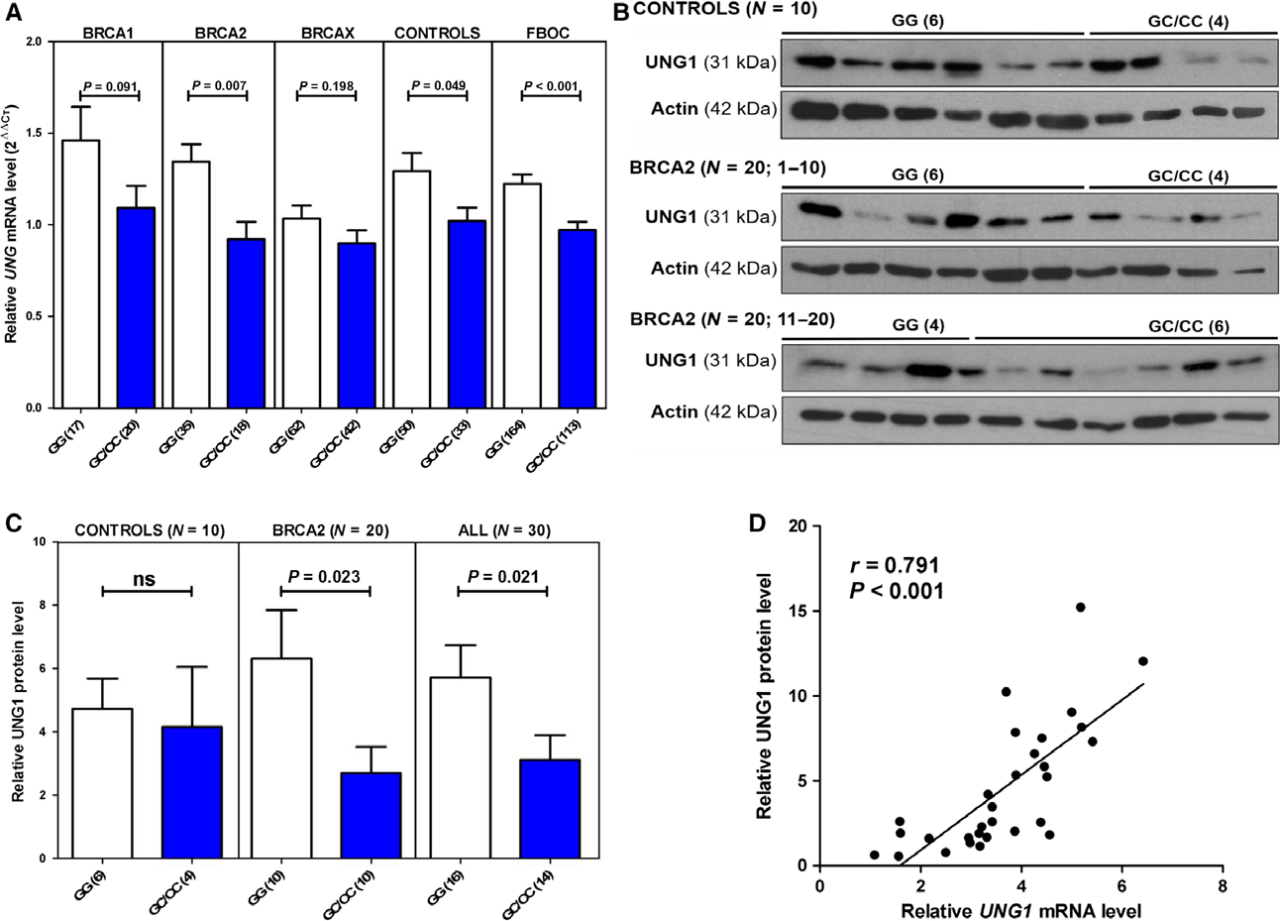
*UNG* mRNA and protein levels. (A) *UNG* mRNA levels in the various FBOC groups according to the presence or absence of the SNP [noncarriers (GG)/carriers (GC/CC)]. (B) UNG1 protein levels in controls (*n* = 10) and *BRCA2* mutation carriers (*n* = 20) according to the presence or absence of the SNP [noncarriers (GG)/carriers (GC/CC)]. Actin levels were used to normalize for protein loading. (C) Quantification of UNG1 protein levels of the western blot shown in (B). Bars show the mean and the standard error of the mean (SEM). Numbers in brackets indicate sample size. (D) Correlation analysis between *UNG1* mRNA and protein levels in the patients shown in (B). Unpaired *t*‐tests were performed for statistical significance in (A) and (C), Spearman's test was used to test the significance of the correlation in panel (D).

In parallel, we analyzed *UNG* mRNA levels in the FBOC series considering the BRCA status and the presence or absence of the *UNG* variant (Fig. 1A). First, we confirmed in a subgroup of samples (*n* = 97) that the mRNA levels of both *UNG* isoforms (UNG1 and UNG2) were highly correlated (*r* = 0.551; *P* < 0.001) and that total *UNG* mRNA expression was correlated with each of the two isoforms and, therefore, representative of both (Fig. S1). We detected significantly lower *UNG* mRNA expression in individuals harboring the variant (effect size = −0.209; *P* < 0.001). The effect was more pronounced in the *BRCA2* group (effect size = −0.366; *P* = 0.007) and remained significant when analyzing both isoforms separately (Fig. S2).

Given that the SNP protective effect is for ovarian cancer, we also determined *UNG* mRNA expression in tissues of 17 prophylactic oophorectomies from *BRCA1* and *BRCA2* mutation carriers. In this cohort, we also found a trend toward lower total *UNG* mRNA expression associated with the studied SNP (*P* = 0.056; Fig. S3), which was significant for the *UNG1* isoform (*P* = 0.045).

To confirm whether this downregulation was translated into lower expression of the protein, we determined UNG1 protein expression by western blotting (WB) in 10 controls, of which 4 were carriers of the SNP, and in 20 *BRCA2* carriers, of which 10 were carriers of the SNP (Fig. 1B). Quantification showed that *BRCA2* carriers harboring SNP rs34259 had lower UNG1 protein levels (*P* = 0.023) when controls and *BRCA2* carriers were combined, and the effect of SNP rs34259 on UNG1 protein levels remained significant (*P* = 0.021; Fig. 1C). *UNG1* mRNA levels correlated significantly with UNG protein levels in these patients (*r* = 0.791; *P* < 0.001) (Fig. 1D). Finally, we performed WB of both UNG1 and UNG2 in a set of 18 LCLs and confirmed that both UNG isoforms remained highly correlated at the protein level (*r* = 0.829; *P* < 0.001; Fig. S4A,B). Despite the reduced sample size, we also found a trend toward lower UNG1 and UNG2 protein levels in the LCL series associated with the *UNG* variant (Fig. S4C).

### 3.3 Accumulation of DNA damage at the telomeres

We analyzed the accumulation of two kinds of lesions: 8‐oxoguanine and uracil, which are detected by FPG and UNG glycosylases, respectively.

#### 3.3.1 SNP rs34259 is associated with lower oxidative DNA damage

**Figure 2:**
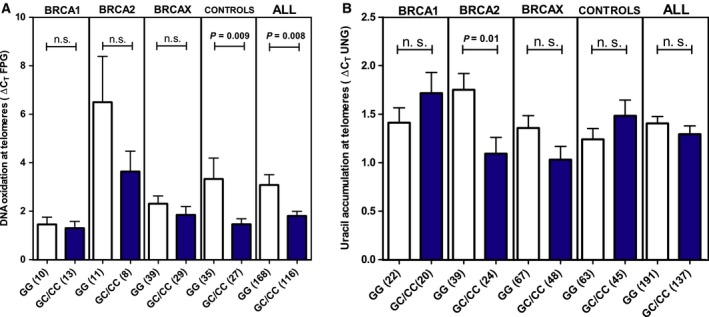
Telomere DNA damage in the various FBOC groups according to the presence or absence of the *UNG* SNP. (A) DNA oxidation at telomeres. (B) Detection of uracil at telomeres in FBOC patients. Bars show the mean and the SEM. Numbers in brackets indicate sample size. Mann–Whitney *U*‐test was used in (A), unpaired *t*‐test was used in (B).

When analyzing the accumulation of 8‐oxoguanine, we observed significantly lower oxidation levels in individuals harboring the variant (*P* = 0.008) (Fig. 2A). We were not able to detect significant differences within each mutational group. However, a statistically significant lower oxidative DNA damage associated with the SNP was found in controls (*P* = 0.009), suggesting that the SNP is associated with lower oxidative damage accumulation at telomeres independently of the *BRCA* status.

#### 3.3.2 *BRCA2* mutation carriers harboring SNP rs34259 show lower uracil accumulation at the telomeres

After treatment with UNG, telomeric DNA showed an average decrease of 54% in PCR amplification compared to a 22% decrease observed when amplifying the 36B4 control locus (*P* < 0.0001), reflecting a predominant presence of uracil in telomeres (Fig. S5).

We did not find significant differences in uracil levels at telomeres among BRCA groups or controls. However, when we stratified according to the SNP (Fig. 2B), we detected a significantly lower uracil accumulation at telomeres when the variant was present for *BRCA2* mutation carriers (*P* = 0.01). This result suggests that the protective effect for ovarian cancer risk associated with SNP rs34259 in *BRCA2* mutation carriers could be due to an increased UNG activity, leading to less accumulation of uracil at the telomere region.

### 3.4 Lower protein carbonylation level in individuals harboring SNP rs34259

**Figure 3:**
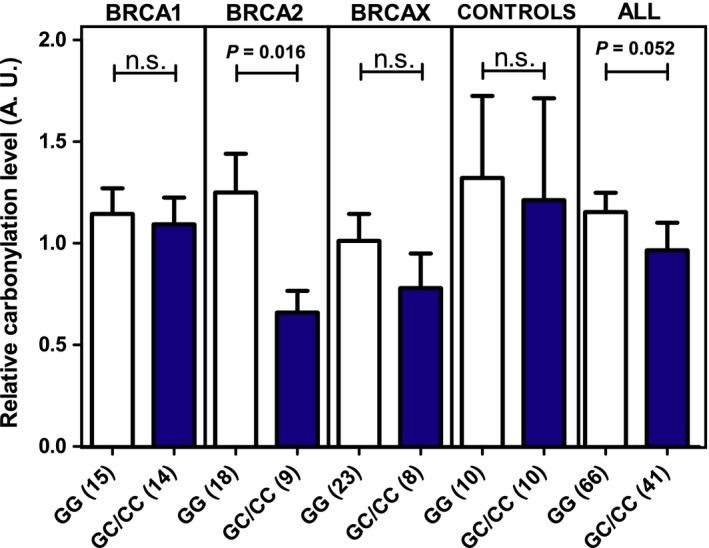
Immunodetection of protein‐bound carbonyl groups in plasma samples from the FBOC series. Carbonylation levels in the different groups stratified according to the presence or absence of SNP rs34259 in *UNG* [noncarriers (GG)/carriers (GC/CC)]. Bars show the mean and the SEM. Numbers in brackets indicate sample size. Unpaired *t*‐tests were performed for statistical significance. A.U., arbitrary units.

No significant differences were found in carbonylation levels in relation to the BRCA status. Notwithstanding, we found a trend toward lower carbonylation levels in all FBOC individuals with the variant (*P* = 0.052). In addition, for *BRCA2* mutation carriers harboring the SNP we detected a significantly lower carbonylation level (*P* = 0.016) (Fig. 3). These results suggest that the SNP in *UNG* is associated with lower oxidative stress susceptibility that becomes pronounced in *BRCA2* mutation carriers.

### 3.5 Shorter telomeres in *BRCA2* mutation carriers harboring the SNP

**Figure 4:**
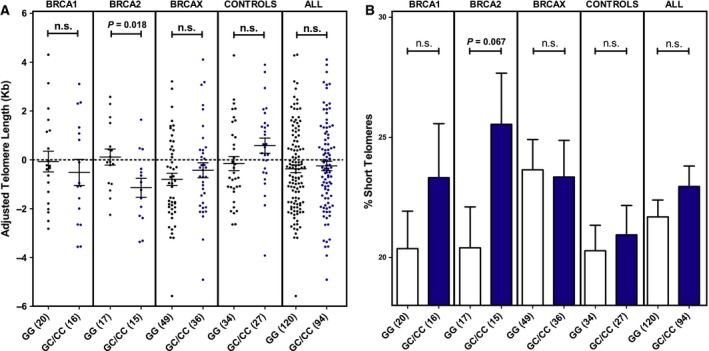
Telomere length and percentage of short telomeres. (A) Distribution of telomere length (kb) values adjusted for age in the FBOC series according to the presence or absence of the *UNG* SNP [noncarriers (GG)/carriers (GC/CC)]. (B) Comparative analysis of FBOC groups regarding the percentage of short (< 3 kb) telomeres. Bars show the mean and the SEM. Numbers in brackets indicate sample size. Unpaired *t*‐tests were performed for statistical significance.

We first evaluated TL distribution in 91 healthy women as a function of age to obtain a regression line to adjust TL in the FBOC samples. As expected, we found a decrease in TL with age (Fig. S6). When the effect of rs34259 was analyzed for each BRCA mutation group, we only found a significant effect among *BRCA2* mutation carriers: In this group, SNP carriers had a reduced age‐adjusted TL (*P* = 0.018; Fig. 4A) and showed a trend toward accumulation of short telomeres (*P* = 0.067; Fig. 4B).

### 3.6 Telomerase activity

We found a significant correlation between telomerase activity and telomere length (*r* = 0.313; *P* < 0.001). Mean telomerase activity was lower when the SNP was present in all groups, but it did not reach statistical significance (Fig. S7).

## 4 Discussion

The SNP rs34259 in the 3′UTR of the *UNG* gene may decrease ovarian cancer risk in *BRCA2* mutation carriers (Osorio *et al*., 2014). However, the molecular mechanism underlying this association is unknown. In the present report, we show that rs34259 is associated with significant *UNG* downregulation and with lower levels of oxidative DNA damage at telomeres. In addition, we found that for *BRCA2* mutation carriers the SNP is associated with significantly lower oxidative stress susceptibility and lower uracil accumulation at telomeres.

As it has been previously demonstrated that the region where the variant is located is a potential seed site for microRNAs that downregulate *UNG* expression (Hegre *et al*., 2013), we decided to explore the effect of this SNP on *UNG* mRNA and protein levels. We detected significantly lower *UNG* mRNA and UNG1 protein levels associated with SNP rs34259, which became pronounced in *BRCA2* mutation carriers. It has been shown that overexpression of human UNG in yeast causes DNA damage due to the generation of AP sites faster than they are repaired (Elder *et al*., 2003). In this regard, the lower UNG expression associated with rs34259 may prevent AP repair from becoming saturated, and this may in part explain its protective effect.

Given the dominant role of UNG for processing uracil at telomeres (Cortizas *et al*., 2016), we evaluated uracil accumulation and observed that this was higher in telomeric DNA than in other genomic regions (Fig. S5), confirming that telomeres are prone to uracil accumulation (Vallabhaneni *et al*., 2015). When we analyzed the SNP effect, we found significantly lower uracil accumulation when the SNP was present, but only for *BRCA2* carriers (Fig. 2B). This suggests that rs34259 could have a positive impact on UNG enzyme performance that may help to explain the protective effect of this SNP in *BRCA2* carriers.

Furthermore, we explored the impact of this SNP on other features related to telomere biology, such as oxidative damage. Because telomeres are especially susceptible to DNA oxidation (O'Callaghan *et al*., 2011; Von Zglinicki *et al*., 2000), we evaluated the accumulation of 8‐oxoguanine as a measure of oxidative damage. We observed significantly lower 8‐oxoguanine levels in individuals harboring the variant (Fig. 2A), suggesting that the SNP is associated with lower oxidative DNA damage accumulation at the telomeres.

We found that the SNP impact on *UNG* expression affects both nuclear (UNG2) and mitochondrial (UNG1) isoforms (Fig. S2). Therefore, apart from the telomeres, it is probable that mitochondrial DNA of patients harboring the SNP presents lower damage, given that oxidative base lesions in mitochondria are repaired by UNG1 (Akbari *et al*., 2007). In addition, we analyzed whether the lower levels of oxidative DNA damage associated with the SNP could be related to lower chronic oxidative stress susceptibility. We found lower protein carbonylation levels when rs34259 was present (Fig. 3), and this was more pronounced in *BRCA2* mutation carriers. These results suggest that the SNP in *UNG* is associated with lower oxidative stress susceptibility, especially for *BRCA2* carriers. Oxidative stress plays an important role in the development and progression of cancer (Valko *et al*., 2006), and therefore, the lower oxidative stress associated with the SNP may help to explain the lower cancer risk of *BRCA2* carriers that harbor the SNP.

We also found a significantly shorter TL associated with the SNP in carriers of *BRCA2* mutations (Fig. 4A). TL is regulated by the shelterin protein complex that protects telomeres (De Lange, 2005) and by telomerase, a ribonucleoprotein complex that adds TTAGGG repeats to the chromosome ends (Blackburn, 2001). Our data reflect this expected positive correlation between TL and telomerase activity. The accumulation of uracil in telomeres weakens the binding affinity of the shelterin component POT1, increasing the accessibility of telomerase. Thus, UNG deficiency causes defective uracil removal that can lead to lengthening of telomeres, as has been demonstrated in mice (Vallabhaneni *et al*., 2015). According to this model, the short telomeres phenotype observed in *BRCA2* carriers harboring the SNP could be due to the lower uracil accumulation at telomeres, also associated with this group, which facilitates shelterin binding.

## 5 Conclusions

We have found that the ovarian cancer risk modifier SNP rs34259 may have a positive impact on UNG enzyme performance and is associated with lower oxidative levels in *BRCA2* carriers, which may explain the cancer‐protective effect attributed to this SNP in this group. Taken together, our findings support the importance of genetic changes in BER pathway genes as modifiers of cancer susceptibility for *BRCA1* and *BRCA2* mutation carriers.

## Conflict of interest

The authors declare no conflict of interest.

## Author contributions

JB and AO contributed to study conception and design. JMB, CB‐B, VF, MU, JLG‐G, and RP involved in acquisition of data. JMB, CB‐B, JB, and AO contributed to analysis and interpretation of data. JMB and CB‐B drafted the manuscript. JB and AO critically revised the manuscript. All authors read and approved the final manuscript.

## Supporting information


**Fig. S1.** (A) Correlation analysis between total *UNG* mRNA expression and *UNG1* mRNA expression. (B) Correlation analysis between total *UNG* mRNA expression and *UNG2* mRNA expression. (C) Correlation analysis between *UNG1* mRNA and *UNG2* mRNA expression. Spearman's test was used to assess the significance of the correlations.

**Fig. S2.** Expression levels of specific isoforms of *UNG* mRNA according to the presence or absence of the SNP (noncarriers (GG)/carriers (GC/CC)).

**Fig. S3.** Expression levels of specific isoforms of *UNG* mRNA according to the presence or absence of the SNP (noncarriers (GG)/carriers (GC/CC)) in ovarian tissue from *BRCA1* and *BRCA2* patients (*n* = 17).

**Fig. S4.** (A) Western blot of UNG1 and UNG2 in a panel of 18 established lymphoblastoid cell lines (LCLs) (Vaclová *et al*., 2015). Briefly, the LCLs were established by Epstein‐Barr virus transformation of peripheral blood lymphocytes from eleven healthy women carrying heterozygous mutations in *BRCA1* and seven noncarrier relatives (controls). None of the women included in the study had personal antecedents of cancer. Cells were cultured in RPMI‐1640 media (Sigma‐Aldrich) supplemented with 20% non‐heat‐inactivated fetal bovine serum (Sigma‐Aldrich), penicillin‐streptomycin (Gibco) and Fungizone (Gibco). Cells were cultured at 37 °C in a 5% CO_2_ atmosphere and were maintained in exponential growth by daily dilution to 10^6^ cells·mL^−1^ complete media. Protein extraction and western blotting were performed as described in the Materials and Methods section. (B) Correlation analysis between UNG1 and UNG2 protein expression levels in LCLs. Spearman's test was used to assess the significance of the correlation. (C) UNG1 and UNG2 expression levels in the LCL series according to the presence or absence of the SNP (noncarriers (GG)/carriers (GC/CC)). Bars show the mean and the standard error of the mean (SEM). Numbers in brackets indicate sample size. Unpaired *t*‐tests were performed for statistical significance. DNA extraction and SNP genotyping were performed as are described in the Materials and Methods section.

**Fig. S5.** PCR amplification efficiency at the untreated and UNG‐treated telomeric and *36B4* loci.

**Fig. S6.** Telomere length (TL) distribution in peripheral blood leukocytes as a function of age for the control population (*n* = 91), measured by HT QFISH.

**Fig. S7.** Comparative analysis of telomerase activity in the FBOC series according to the presence or absence of the *UNG* SNP (noncarriers (GG)/carriers (GC/CC)).

**Table S1.** Primers used for *UNG* RNA expression analysis.

**Table S2.** Linear regression analysis in *BRCA* 1/2 mutation carriers.

**Table S3.** Variants within the block of linkage disequilibrium (LD) > 0.8 with SNP rs34259.

**Table S4.** Frequency distribution of the *UNG* variant rs34259 among FBOC groups.

**Table S5.** Summary of information in the GTEx eQTL server regarding transcriptional downregulation of *UNG* in 16 different tissues when rs34259 is present.

## References

[mol212470-bib-0001] Akbari M , Otterlei M , Peña‐Diaz J and Krokan HE (2007) Different organization of base excision repair of uracil in DNA in nuclei and mitochondria and selective upregulation of mitochondrial uracil‐DNA glycosylase after oxidative stress. Neuroscience 145, 1201–1212. 17101234 10.1016/j.neuroscience.2006.10.010

[mol212470-bib-0002] Amos CI , Dennis J , Wang Z , Byun J , Schumacher FR , Gayther SA , Casey G , Hunter DJ , Sellers TA , Gruber SB *et al* (2017) The Oncoarray Consortium: a network for understanding the genetic architecture of common cancers. Cancer Epidemiol Biomarkers Prev 26, 26–36. 10.1158/1055-9965.EPI-16-0106 PMC5224974 27697780

[mol212470-bib-0003] Ardlie KG , Deluca DS , Segre AV , Sullivan TJ , Young TR , Gelfand ET , Trowbridge CA , Maller JB , Tukiainen T , Lek M *et al* (2015) The Genotype‐Tissue Expression (GTEx) pilot analysis: multitissue gene regulation in humans. Science 348, 648–660. 25954001 10.1126/science.1262110 PMC4547484

[mol212470-bib-0004] Benítez‐Buelga C , Baquero JM , Vaclová T , Fernández V , Martín P , Inglada‐Pérez L , Urioste M , Osorio A and Benítez J (2017) Genetic variation in the NEIL2 DNA glycosylase gene is associated with oxidative DNA damage in BRCA2 mutation carriers. Oncotarget 8, 114626–114636. 29383107 10.18632/oncotarget.22638 PMC5777719

[mol212470-bib-0005] Benítez‐Buelga C , Sanchez‐Barroso L , Gallardo M , Apellániz‐Ruiz M , Inglada‐Pérez L , Yanowski K , Carrillo J , Garcia‐Estevez L , Calvo I , Perona R *et al* (2015) Impact of chemotherapy on telomere length in sporadic and familial breast cancer patients. Breast Cancer Res Treat 149, 385–394. 25528024 10.1007/s10549-014-3246-6 PMC4824277

[mol212470-bib-0006] Benítez‐Buelga C , Vaclová T , Ferreira S , Urioste M , Inglada‐Pérez L , Soberón N , Blasco M , Osorio A and Benitez J (2016) Molecular insights into the OGG1 gene, a cancer risk modifier in BRCA1 and BRCA2 mutations carriers. Oncotarget 7, 25815–25825. 27015555 10.18632/oncotarget.8272 PMC5041946

[mol212470-bib-0007] Blackburn EH (2001) Switching and signaling at the telomere. Cell 106, 661–673. 11572773 10.1016/s0092-8674(01)00492-5

[mol212470-bib-0008] Bryant HE , Schultz N , Thomas HD , Parker KM , Flower D , Lopez E , Kyle S , Meuth M , Curtin NJ and Helleday T (2005) Specific killing of BRCA2‐deficient tumours with inhibitors of poly (ADP‐Ribose) polymerase. Nature 434, 913–917. 15829966 10.1038/nature03443

[mol212470-bib-0009] Canela A , Vera E , Klatt P and Blasco MA (2007) High‐throughput telomere length quantification by FISH and its application to human population studies. Proc Natl Acad Sci USA 104, 5300–5305. 17369361 10.1073/pnas.0609367104 PMC1828130

[mol212470-bib-0010] Carithers LJ , Ardlie K , Barcus M , Branton PA , Britton A , Buia SA , Compton CC , DeLuca DS , Peter‐Demchok J , Gelfand ET *et al* (2015) A novel approach to high‐quality postmortem tissue procurement: the GTEx project. Biopreserv Biobank 13, 311–319. 26484571 10.1089/bio.2015.0032 PMC4675181

[mol212470-bib-0011] Cortizas EM , Zahn A , Safavi S , Reed JA , Vega F , Di Noia JM and Verdun RE (2016) UNG protects B cells from AID‐induced telomere loss. J Exp Med 213, 2459–2472. 27697833 10.1084/jem.20160635 PMC5068241

[mol212470-bib-0012] De Lange T (2005) Shelterin: the protein complex that shapes and safeguards human telomeres. Genes Dev 19, 2100–2110. 16166375 10.1101/gad.1346005

[mol212470-bib-0013] Elder RT , Zhu X , Priet S , Chen M , Yu M , Navarro JM , Sire J and Zhao Y (2003) A fission yeast homologue of the human Uracil‐DNA‐glycosylase and their roles in causing DNA damage after overexpression. Biochem Biophys Res Commun 306, 693–700. 12810074 10.1016/s0006-291x(03)01036-2

[mol212470-bib-0016] Farmer H , McCabe N , Lord CJ , Tutt ANJ , Johnson DA , Richardson TB , Santarosa M , Dillon KJ , Hickson I , Knights C *et al* (2005) Targeting the DNA repair defect in BRCA mutant cells as a therapeutic strategy. Nature 434, 917–921. 15829967 10.1038/nature03445

[mol212470-bib-0014] Fedorova M , Bollineni RC and Hoffmann R (2013) Protein carbonylation as a major hallmark of oxidative damage: update of analytical strategies. Mass Spectrom Rev 33, 79–97. 23832618 10.1002/mas.21381

[mol212470-bib-0015] García‐Giménez JL , Velázquez‐Ledesma AM , Esmoris I , Romá‐Mateo C , Sanz P , Viña J and Federico Pallardó FV (2012) Histone carbonylation occurs in proliferating cells. Free Radic Biol Med 52, 1453–1464. 22342519 10.1016/j.freeradbiomed.2012.01.022

[mol212470-bib-0017] Hegde ML , Hazra TK and Mitra S (2008) Early steps in the DNA base excision/single‐strand interruption repair pathway in mammalian cells. Cell Res 18, 27–47. 18166975 10.1038/cr.2008.8 PMC2692221

[mol212470-bib-0018] Hegre SA , Sætrom P , Aas PA , Pettersen HS , Otterlei M and Krokan HE (2013) Multiple microRNAs may regulate the DNA repair enzyme uracil‐DNA glycosylase. DNA Repair 12, 80–86. 23228472 10.1016/j.dnarep.2012.10.007

[mol212470-bib-0019] Jia P , Chengtao H and Chai W (2015) DNA excision repair at telomeres. DNA Repair 36, 137–145. 26422132 10.1016/j.dnarep.2015.09.017 PMC4688237

[mol212470-bib-0020] Krokan HE , Drabløs F and Slupphaug G (2002) Uracil in DNA – occurrence, consequences and repair. Oncogene 21, 8935–8948. 12483510 10.1038/sj.onc.1205996

[mol212470-bib-0021] Maynard S , Schurman SH , Harboe C , de Souza‐Pinto NC and Bohr VA (2009) Base excision repair of oxidative DNA damage and association with cancer and aging. Carcinogenesis 30, 2–10. 18978338 10.1093/carcin/bgn250 PMC2639036

[mol212470-bib-0022] Milne RL , Osorio A , Cajal TR , Vega A , Llort G , De La Hoya M , Díez O , Alonso MC , Lazaro C , Blanco I *et al* (2008) The average cumulative risks of breast and ovarian cancer for carriers of mutations in BRCA1 and BRCA2 attending genetic counseling units in Spain. Clin Cancer Res 14, 2861–2869. 18451254 10.1158/1078-0432.CCR-07-4436

[mol212470-bib-0023] Nilsen H , Otterlei M , Haug T , Solum K , Nagelhus TA , Skorpen F and Krokan HE (1997) Nuclear and mitochondrial uracil‐DNA glycosylases are generated by alternative splicing and transcription from different positions in the UNG gene. Nucleic Acids Res 25, 750–755. 9016624 10.1093/nar/25.4.750 PMC146498

[mol212470-bib-0024] O'Callaghan N , Baack N , Sharif R and Fenech M (2011) A qPCR‐based assay to quantify oxidized guanine and other FPG‐sensitive base lesions within telomeric DNA. Biotechniques 51, 403–412. 22150331 10.2144/000113788

[mol212470-bib-0025] O'Donovan PJ and Livingston DM (2010) BRCA1 and BRCA2: breast/ovarian cancer susceptibility gene products and participants in DNA double‐strand break repair. Carcinogenesis 31, 961–967. 20400477 10.1093/carcin/bgq069

[mol212470-bib-0026] Osorio A , Milne RL , Kuchenbaecker K , Vaclová T , Pita G , Alonso R , Peterlongo P , Blanco I , de la Hoya M , Duran M *et al* (2014) DNA glycosylases involved in base excision repair may be associated with cancer risk in BRCA1 and BRCA2 mutation carriers. PLoS Genet 10, e1004256. 24698998 10.1371/journal.pgen.1004256 PMC3974638

[mol212470-bib-0027] Pooley KA , Bojesen SE , Weischer M , Nielsen SF , Thompson D , Olama AA , Michailidou K , Tyrer JP , Benlloch S , Brown J *et al* (2013) A Genome‐Wide Association Scan (GWAS) for mean telomere length within the COGS project: identified loci show little association with hormone‐related cancer risk. Hum Mol Genet 22, 5056–5064. 23900074 10.1093/hmg/ddt355 PMC3836481

[mol212470-bib-0028] Vaclová T , Gómez‐López G , Setién F , Garcia‐Bueno JM , Macias JA , Barroso A , Urioste M , Esteller M , Benítez J and Osorio A (2015) DNA repair capacity is impaired in healthy BRCA1 heterozygous mutation carriers. Breast Cancer Res Treat 152, 271–282. 26071757 10.1007/s10549-015-3459-3

[mol212470-bib-0029] Valko M , Rhodes CJ , Moncol J , Izakovic M and Mazur M (2006) Free radicals, metals and antioxidants in oxidative stress‐induced cancer. Chem Biol Interact 160, 1–40. 16430879 10.1016/j.cbi.2005.12.009

[mol212470-bib-0030] Vallabhaneni H , Zhou F , Maul RW , Sarkar J , Yin J , Lei M , Harrington L , Gearhart PJ and Liu Y (2015) Defective repair of uracil causes telomere defects in mouse hematopoietic cells. J Biol Chem 290, 5502–5511. 25572391 10.1074/jbc.M114.607101 PMC4342465

[mol212470-bib-0031] Von Zglinicki T , Pilger R and Sitte N (2000) Accumulation of single‐strand breaks is the major cause of telomere shortening in human fibroblasts. Free Radic Biol Med 28, 4–74. 10.1016/s0891-5849(99)00207-5 10656292

[mol212470-bib-0032] Ward LD and Kellis M (2012) HaploReg: a resource for exploring chromatin states, conservation, and regulatory motif alterations within sets of genetically linked variants. Nucleic Acids Res 40, 930–934. 10.1093/nar/gkr917 PMC3245002 22064851

[mol212470-bib-0033] Westra HJ , Peters MJ , Esko T , Yaghootkar H , Schurmann C , Kettunen J , Christiansen MW , Fairfax BP , Schramm K , Powell JE *et al* (2013) Systematic identification of trans eQTLs as putative drivers of known disease associations. Nat Genet 45, 1238–1243. 24013639 10.1038/ng.2756 PMC3991562

[mol212470-bib-0034] Xue JH , Xu GF , Gu TP , Chen GD , Han BB , Xu ZM , Bjørås M , Krokan HE , Xu GL and Du YR (2016) Uracil‐DNA glycosylase UNG promotes Tet‐mediated DNA demethylation. J Biol Chem 291, 731–738. 26620559 10.1074/jbc.M115.693861 PMC4705393

[mol212470-bib-0035] Zerbino DR , Achuthan P , Akanni W , Amode MR , Barrell D , Bhai J , Billis K , Cummins C , Gall A , Girón CG *et al* (2018) Ensembl 2018. Nucleic Acids Res 46, 754–761. 10.1093/nar/gkx1098 PMC5753206 29155950

[mol212470-bib-0036] Zharkov DO , Mechetin GV and Nevinsky GA (2010) Uracil‐DNA glycosylase: structural, thermodynamic and kinetic aspects of lesion search and recognition. Mutat Res 685, 11–20. 19909758 10.1016/j.mrfmmm.2009.10.017 PMC3000906

