# Supplementary material for: A common SNP in the UNG gene decreases ovarian cancer risk in BRCA2 mutation carriers
Source: Mol Oncol. 2019 Mar 1;13(5):1110–20. doi: 10.1002/1878-0261.12470 (PMC6487686; doi:10.1002/1878-0261.12470)
Supplement: Supplementary file 1 — Fig. S1. (A) Correlation analysis between total UNG mRNA expression and UNG1 mRNA expression. (B) Correlation analysis between total UNG mRNA expression and UNG2 mRNA expression. (C) Correlation analysis between UNG1 mRNA and UNG2 mRNA expression. Spearman's test was used to assess the significance of the correlations. [file MOL2-13-1110-s001.docx]

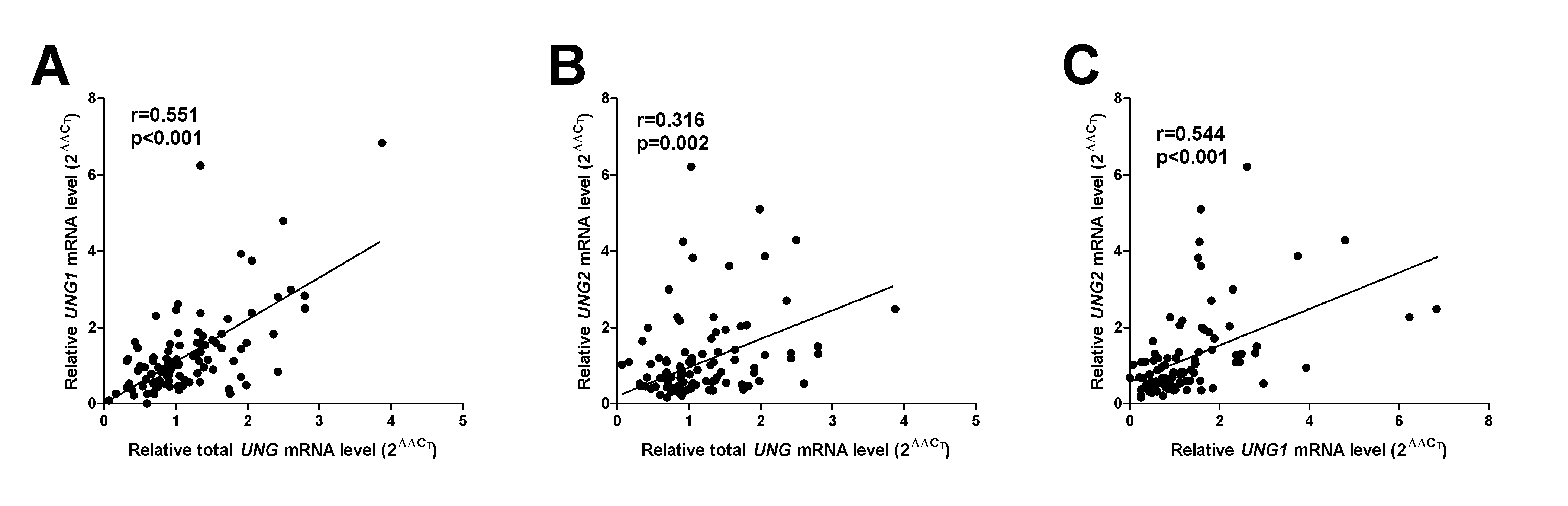


**Figure S1.** A) Correlation analysis between total *UNG* mRNA expression and *UNG1* mRNA expression. B) Correlation analysis between total *UNG* mRNA expression and *UNG2* mRNA expression. C) Correlation analysis between *UNG1* mRNA and *UNG2* mRNA expression. Spearman's test was used to assess the significance of the correlations.
