# Supplementary material for: A common SNP in the UNG gene decreases ovarian cancer risk in BRCA2 mutation carriers
Source: Mol Oncol. 2019 Mar 1;13(5):1110–20. doi: 10.1002/1878-0261.12470 (PMC6487686; doi:10.1002/1878-0261.12470)
Supplement: Supplementary file 2 — Fig. S2. Expression levels of specific isoforms of UNG mRNA according to the presence or absence of the SNP (noncarriers (GG)/carriers (GC/CC)). [file MOL2-13-1110-s002.docx]

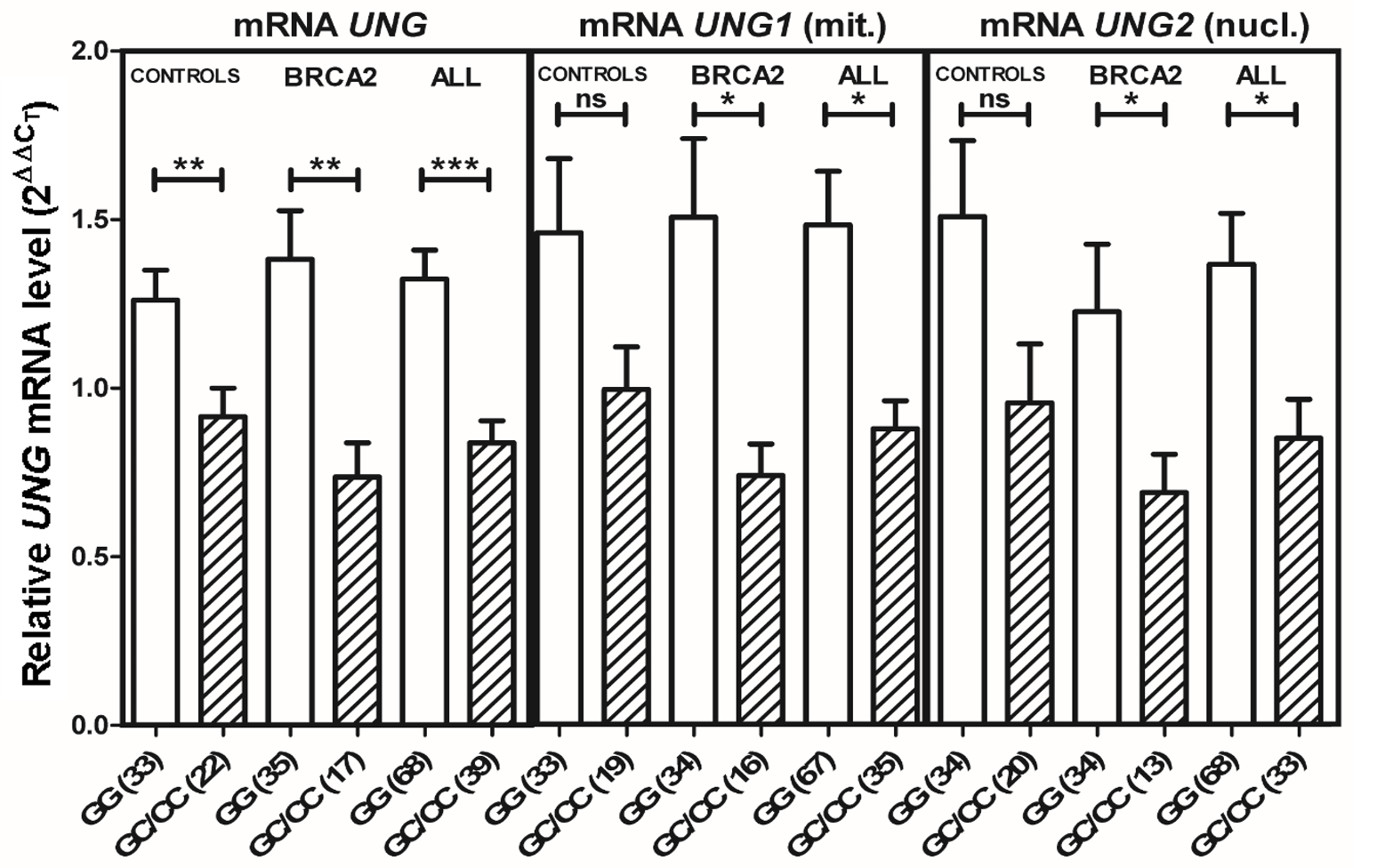


**Figure S2.** Expression levels of specific isoforms of *UNG* mRNA according to the presence or absence of the SNP (non-carriers (GG)/carriers (GC/CC)). Bars show the mean and the SEM. Numbers in brackets denote sample size. Unpaired *t*‐tests were performed for statistical significance (*p<0.05, ** p<0.01, ***p<0.001).
