# Supplementary material for: A common SNP in the UNG gene decreases ovarian cancer risk in BRCA2 mutation carriers
Source: Mol Oncol. 2019 Mar 1;13(5):1110–20. doi: 10.1002/1878-0261.12470 (PMC6487686; doi:10.1002/1878-0261.12470)
Supplement: Supplementary file 3 — Fig. S3. Expression levels of specific isoforms of UNG mRNA according to the presence or absence of the SNP (noncarriers (GG)/carriers (GC/CC)) in ovarian tissue from BRCA1 and BRCA2 patients (n = 17). [file MOL2-13-1110-s003.docx]

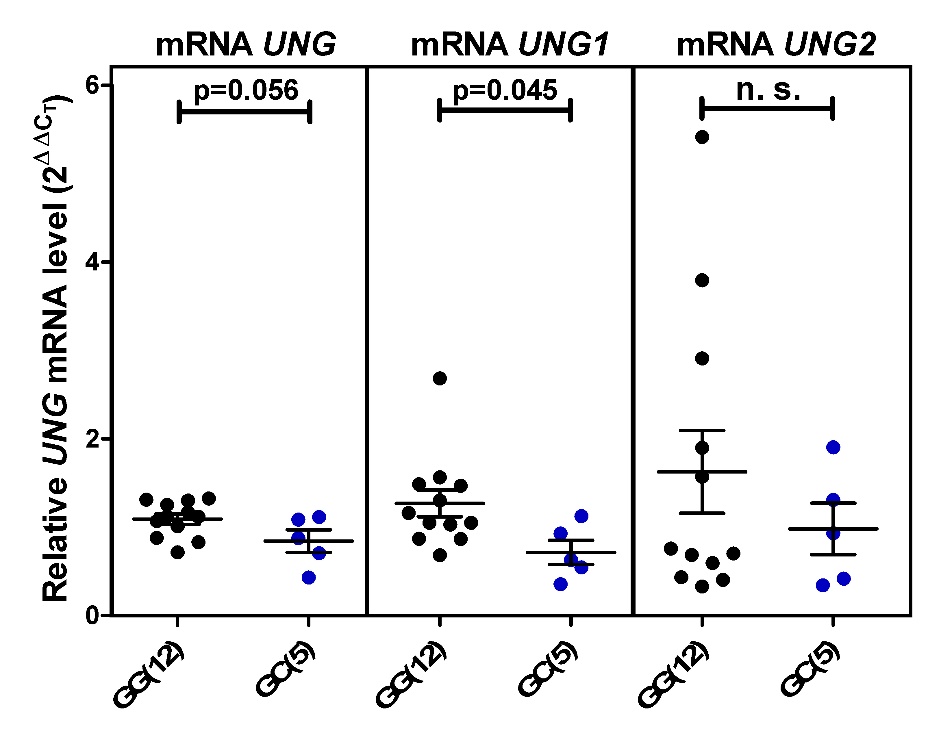


**Figure S3.** Expression levels of specific isoforms of *UNG* mRNA according to the presence or absence of the SNP (non-carriers (GG)/carriers (GC/CC)) in ovarian tissue from *BRCA1* and *BRCA2* patients (n=17). Twenty mg of ovarian tissue from ovarian biopsies preserved in OCT were resuspended in TRIzol® Reagent (Thermo Fisher Scientific) and were homogenized using a Precellys® 24 tissue homogenizer (Bertin Instruments). DNA was extracted using the DNeasy® Blood & Tissue Kit (Qiagen) following the manufacturer’s instructions. RNA extraction, cDNA synthesis, qPCR analysis and SNP genotyping were performed as described in the Materials and Methods section. Bars show the mean and the SEM. Numbers in brackets denote sample size. Unpaired *t*‐tests were performed for statistical significance.
