# Supplementary material for: A common SNP in the UNG gene decreases ovarian cancer risk in BRCA2 mutation carriers
Source: Mol Oncol. 2019 Mar 1;13(5):1110–20. doi: 10.1002/1878-0261.12470 (PMC6487686; doi:10.1002/1878-0261.12470)
Supplement: Supplementary file 4 — Fig. S4. (A) Western blot of UNG1 and UNG2 in a panel of 18 established lymphoblastoid cell lines (LCLs) (Vaclová et al., 2015). Briefly, the LCLs were established by Epstein‐Barr virus transformation of peripheral blood lymphocytes from eleven healthy women carrying heterozygous mutations in BRCA1 and seven noncarrier relatives (controls). None of the women included in the study had personal antecedents of cancer. Cells were cultured in RPMI‐1640 media (Sigma‐Aldrich) supplemented with 20% non‐heat‐inactivated fetal bovine serum (Sigma‐Aldrich), penicillin‐streptomycin (Gibco) and Fungizone (Gibco). Cells were cultured at 37 °C in a 5% CO2 atmosphere and were maintained in exponential growth by daily dilution to 106 cells·mL−1 complete media. Protein extraction and western blotting were performed as described in the Materials and Methods section. (B) Correlation analysis between UNG1 and UNG2 protein expression levels in LCLs. Spearman's test was used to assess the significance of the correlation. (C) UNG1 and UNG2 expression levels in the LCL series according to the presence or absence of the SNP (noncarriers (GG)/carriers (GC/CC)). Bars show the mean and the standard error of the mean (SEM). Numbers in brackets indicate sample size. Unpaired t‐tests were performed for statistical significance. DNA extraction and SNP genotyping were performed as are described in the Materials and Methods section. [file MOL2-13-1110-s004.docx]

**
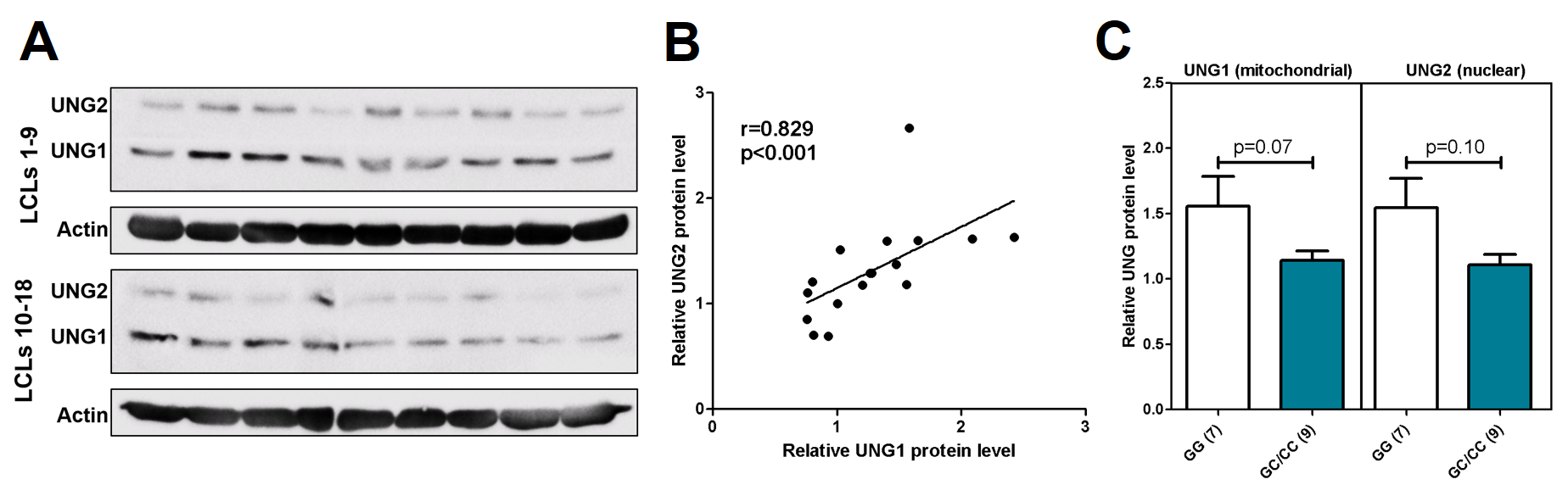
**

**Figure S4.** (A) Western blot of UNG1 and UNG2 in a panel of 18 established lymphoblastoid cell lines (LCLs)^15^. Briefly, the LCLs were established by Epstein Barr virus transformation of peripheral blood lymphocytes from eleven healthy women carrying heterozygous mutations in *BRCA1* and seven non-carrier relatives (controls). None of the women included in the study had personal antecedents of cancer. Cells were cultured in RPMI-1640 media (Sigma-Aldrich) supplemented with 20% non-heat-inactivated fetal bovine serum (Sigma-Aldrich), penicillin-streptomycin (Gibco) and Fungizone (Gibco). Cells were cultured at 37°C in a 5% CO2 atmosphere and were maintained in exponential growth by daily dilution to 10^6^ cells/ml complete media. Protein extraction and western blotting were performed as described in the Materials and Methods section. (B) Correlation analysis between UNG1 and UNG2 protein expression levels in LCLs. Spearman's test was used to assess the significance of the correlation. (C) UNG1 and UNG2 expression levels in the LCL series according to the presence or absence of the SNP (non-carriers (GG)/carriers (GC/CC)). Bars show the mean and the standard error of the mean (SEM). Numbers in brackets indicate sample size. Unpaired *t*‐tests were performed for statistical significance. DNA extraction and SNP genotyping were performed as are described in the Materials and Methods section.
