# Supplementary material for: A common SNP in the UNG gene decreases ovarian cancer risk in BRCA2 mutation carriers
Source: Mol Oncol. 2019 Mar 1;13(5):1110–20. doi: 10.1002/1878-0261.12470 (PMC6487686; doi:10.1002/1878-0261.12470)
Supplement: Supplementary file 5 — Fig. S5. PCR amplification efficiency at the untreated and UNG‐treated telomeric and 36B4 loci. [file MOL2-13-1110-s005.docx]

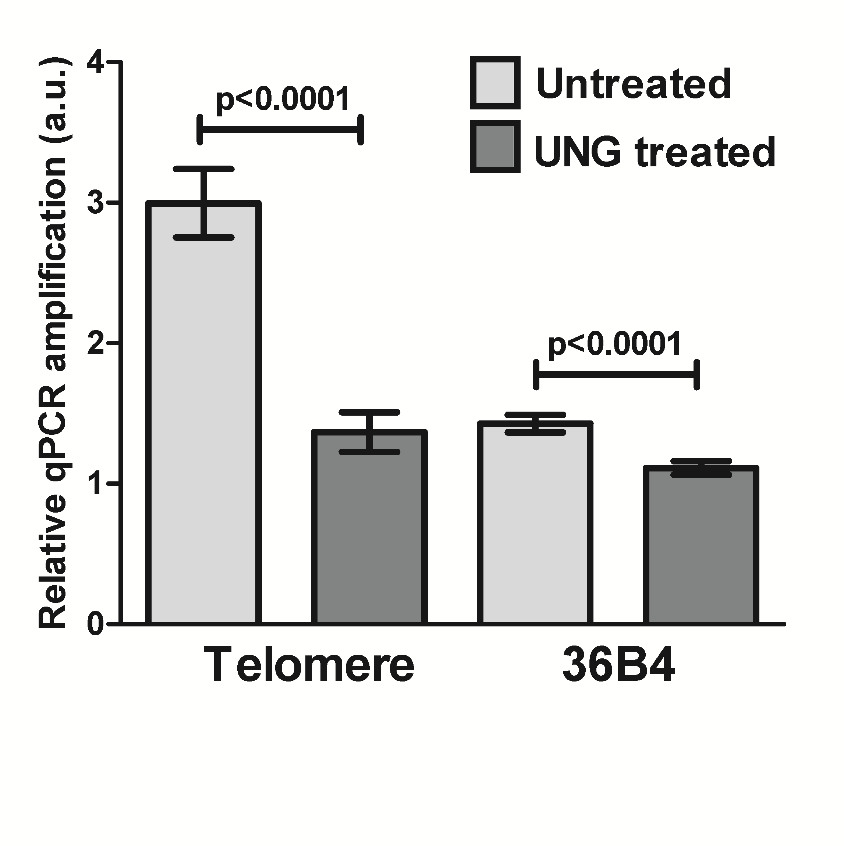


**Figure S5.** PCR amplification efficiency at the untreated and UNG-treated telomeric and *36B4* loci. qPCR amplification efficiency in the untreated/treated samples was normalized to the average expression level for each target genomic region. Since no significant differences between BRCA groups were found, all FBOC cases were grouped together (n=328). Bars show the mean + standard error of the mean (SEM). Unpaired *t*‐tests were performed for statistical significance. a. u.: arbitrary units.
