# Supplementary material for: A common SNP in the UNG gene decreases ovarian cancer risk in BRCA2 mutation carriers
Source: Mol Oncol. 2019 Mar 1;13(5):1110–20. doi: 10.1002/1878-0261.12470 (PMC6487686; doi:10.1002/1878-0261.12470)
Supplement: Supplementary file 6 — Fig. S6. Telomere length (TL) distribution in peripheral blood leukocytes as a function of age for the control population (n = 91), measured by HT QFISH. [file MOL2-13-1110-s006.docx]

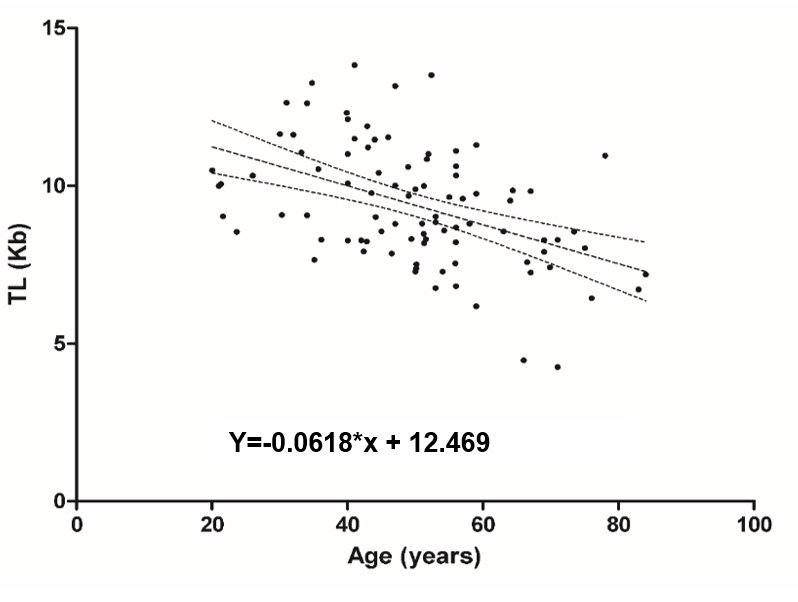


**Figure S6.** Telomere length (TL) distribution in peripheral blood leukocytes as a function of age for the control population (n=91), measured by HT QFISH. The regression line is shown (y= -0.0618 x age + 12.469; r^2^=0.212; p<0.0001).
