# Supplementary material for: A common SNP in the UNG gene decreases ovarian cancer risk in BRCA2 mutation carriers
Source: Mol Oncol. 2019 Mar 1;13(5):1110–20. doi: 10.1002/1878-0261.12470 (PMC6487686; doi:10.1002/1878-0261.12470)
Supplement: Supplementary file 7 — Fig. S7. Comparative analysis of telomerase activity in the FBOC series according to the presence or absence of the UNG SNP (noncarriers (GG)/carriers (GC/CC)). [file MOL2-13-1110-s007.docx]

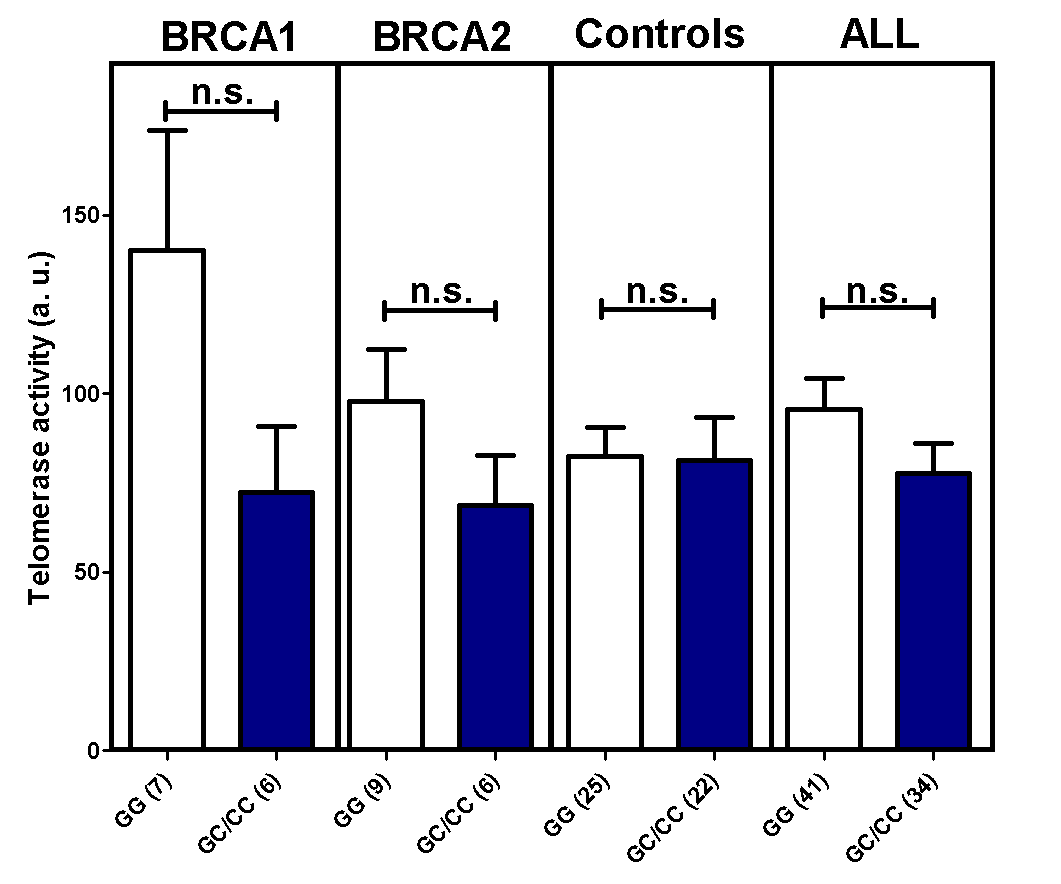


**Figure S7.** Comparative analysis of telomerase activity in the FBOC series according to the presence or absence of the *UNG* SNP (non-carriers (GG)/carriers (GC/CC)). Bars show the mean and the SEM. Numbers in brackets denote sample size. Unpaired t‐tests were performed for statistical significance. a. u.: arbitrary units.
