# Supplementary material for: A common SNP in the UNG gene decreases ovarian cancer risk in BRCA2 mutation carriers
Source: Mol Oncol. 2019 Mar 1;13(5):1110–20. doi: 10.1002/1878-0261.12470 (PMC6487686; doi:10.1002/1878-0261.12470)
Supplement: Supplementary file 8 — Table S1. Primers used for UNG RNA expression analysis. [file MOL2-13-1110-s008.docx]

| Supplementary Table S1: Primers used for *UNG* RNA expression analysis | |
| --- | --- |
| cDNA-*UNG*-F: | 5’ TTGTTCATCCTGGCCATGGA 3’ |
| cDNA-*UNG*-R: | 5’ ACTGCCCTTCTTCTGAGCAT 3’ |
| cDNA-*UNG1*-F: | 5’ ATGGGCGTCTTCTGCCTTG 3’ |
| cDNA-*UNG1*-R: | 5’ CTCTGGATCCGGTCCAACTG 3’ |
| cDNA-*UNG2*-F: | 5’ CCTCCTCAGCTCCAGGATGA 3’ |
| cDNA-*UNG2*-R: | 5’ TCGCTTCCTGGCGGG 3’ |
| cDNA-*GAPDH*-F: | 5’ CCTGCACCACCAACTGCTTA 3’ |
| cDNA-*GAPDH*-R: | 5’ CCATCACGCCACAGTTTCC 3’ |
