# Supplementary material for: A common SNP in the UNG gene decreases ovarian cancer risk in BRCA2 mutation carriers
Source: Mol Oncol. 2019 Mar 1;13(5):1110–20. doi: 10.1002/1878-0261.12470 (PMC6487686; doi:10.1002/1878-0261.12470)
Supplement: Supplementary file 9 — Table S2. Linear regression analysis in BRCA 1/2 mutation carriers. [file MOL2-13-1110-s009.docx]

| Supplementary Table S2: Linear regression analysis in BRCA 1/2 mutation carriers*^a^* | | | | |
| --- | --- | --- | --- | --- |
| Dependent variables | Independent variables | β coefficient*^b^* | p-value*^c^* | 95% C. I. ((Lower) - (Upper limit)) |
| *UNG* mRNA expression | SNP | -0.292 | **0.005** | ((-0.585)-(-0.111)) |
|  | Cancer | 0.029 | 0.773 | ((-0.269)-(0.201)) |
| Adjusted TL | SNP | -0.239 | 0.053 | ((-1.701)-(0.010)) |
|  | Cancer | -0.109 | 0.373 | ((-1.244)-(0.473)) |
| % Short telomeres | SNP | 0.266 | **0.028** | ((0.472)-(7.869)) |
|  | Cancer | 0.209 | 0.081 | ((-0.422)-(7.002)) |
| Telomere oxidation | SNP | -0.250 | 0.106 | ((-4.126)-(0.687)) |
|  | Cancer | -0.218 | 0.156 | ((-4.381)-(0.437)) |
| Uracil at telomeres | SNP | -0.130 | 0.190 | ((-0.621)-(0.125)) |
|  | Cancer | -0.014 | 0.889 | ((-0.393)-(0.341)) |
| Telomerase activity | SNP | -0.111 | 0.395 | ((-41.430)-(16.597)) |
|  | Cancer | -0.237 | 0.072 | ((-55.006)-(2.415)) |
| Carbonylation | SNP | -0.204 | 0.123 | ((-0.574)-(0.071)) |
|  | Cancer | -0.234 | 0.078 | ((-0.033)-(0.605)) |
| *^a^UNG* mRNA expression; adjusted TL (Kb); percentage of short telomeres; telomere oxidation; uracil at telomeres; telomerase activity; and protein carbonylation were included as dependent variables. The SNP and cancer status were included as independent variables. *^b^*β coefficients quantify how much the 2 independent variables (*UNG* SNP and cancer status) modify the dependent variables. *^c^*Unpaired *t*-test was used to check the significance of individual regression coefficients in the multiple linear regression model | | | | |
|  |  |  |  |  |
