# Supplementary material for: A common SNP in the UNG gene decreases ovarian cancer risk in BRCA2 mutation carriers
Source: Mol Oncol. 2019 Mar 1;13(5):1110–20. doi: 10.1002/1878-0261.12470 (PMC6487686; doi:10.1002/1878-0261.12470)
Supplement: Supplementary file 10 — Table S3. Variants within the block of linkage disequilibrium (LD) > 0.8 with SNP rs34259. [file MOL2-13-1110-s010.docx]

| Supplementary Table S3: Variants within the block of linkage disequilibrium (LD) > 0.8 with SNP rs34259 | | | | | | | |
| --- | --- | --- | --- | --- | --- | --- | --- |
| Variant | Class | Ref | Alt | Position | Gene | Location*^a^* | r^2^ (LD) |
| rs34259 | **SNP** | **G** | **C** | **12:109113428** | ***UNG*** | **Downstream – 3' UTR** |  |
| rs34261 | SNP | G | A | 12:109114490 | *UNG* | Downstream – 3' UTR | 0.971065 |
| rs34262 | SNP | C | T | 12:109114670 | *UNG* | Downstream – 3' UTR | 0.971065 |
| rs34263 | SNP | A | G | 12:109115044 | *UNG* | Downstream – 3' UTR | 1 |
| rs2436630 | SNP | G | A | 12:109124655 | *UNG* | Downstream – 3' UTR | 0.918382 |
| rs1610925 | Insertion | - | TA | 12:109099618/9 | *UNG* | Intronic | 0.824795 |
| *^a^*In some cases, the SNP can reside in more than one location, depending on the isoform of the gene. Only one gene location is shown in the table. | | | | | | | |
