# Supplementary material for: A common SNP in the UNG gene decreases ovarian cancer risk in BRCA2 mutation carriers
Source: Mol Oncol. 2019 Mar 1;13(5):1110–20. doi: 10.1002/1878-0261.12470 (PMC6487686; doi:10.1002/1878-0261.12470)
Supplement: Supplementary file 11 — Table S4. Frequency distribution of the UNG variant rs34259 among FBOC groups. [file MOL2-13-1110-s011.docx]

| Supplementary Table S4: Frequency distribution of the UNG variant rs34259 among FBOC groups | | | | | | | | |
| --- | --- | --- | --- | --- | --- | --- | --- | --- |
|  | Allele Frequency | | | Genotype Frequency | | | | |
|  | G | C | p-value*^b^* | GG | GC | CC | GC/CC | p-value*^b^* |
| IBS*^a^* | 171 (79.9%) | 43 (20.1%) |  | 69 (64.5%) | 33 (30.8%) | 5 (4.7%) | 38 (34.0%) |  |
| BRCA1 | 75 (73.5%) | 27 (26.5%) | 0.2018 | 25 (49.0%) | 25 (49.0%) | 1 (2.0%) | 26 (51.0%) | 0.0943 |
| BRCA2 | 96 (77.4%) | 28 (22.6%) | 0.5885 | 39 (62.9%) | 18 (29.0%) | 5 (8.1%) | 23 (37.1%) | 0.8402 |
| BRCA1/2 | 171 (75.7%) | 55 (24.3%) | 0.2850 | 64 (56.6%) | 43 (38.1%) | 6 (5.3%) | 49 (43.4%) | 0.5620 |
| BRCAX | 186 (77.5%) | 54 (22.5%) | 0.5323 | 70 (58.3%) | 46 (38.3%) | 4 (3.3%) | 50 (41.7%) | 0.5888 |
| CONTROLS | 167 (75.2%) | 55 (24.8%) | 0.2417 | 65 (58.6%) | 37 (33.3%) | 9 (8.1%) | 46 (41.4%) | 0.6196 |
| FBOC | 524 (76.2%) | 164 (23.8%) | 0.2553 | 199 (57.8%) | 126 (36.6%) | 19 (5.5%) | 145 (42.2%) | 0.5146 |
| *^a^*Set of samples of the Iberian Populations in Spain of the 1000 Genomes Project Phase 3  *^b^*χ² vs IBS | | | | | | | | |
