# Supplementary material for: A common SNP in the UNG gene decreases ovarian cancer risk in BRCA2 mutation carriers
Source: Mol Oncol. 2019 Mar 1;13(5):1110–20. doi: 10.1002/1878-0261.12470 (PMC6487686; doi:10.1002/1878-0261.12470)
Supplement: Supplementary file 12 — Table S5. Summary of information in the GTEx eQTL server regarding transcriptional downregulation of UNG in 16 different tissues when rs34259 is present. [file MOL2-13-1110-s012.docx]

| Supplementary Table S5: Summary of information in the GTEx eQTL server regarding transcriptional downregulation of *UNG* in 16 different tissues when rs34259 is present | | | | | |
| --- | --- | --- | --- | --- | --- |
| Gene | *^a^*p-Value | rs34259 Effect Size | T-Statistic | Standard Error | Tissue |
| *UNG* | 0.004 | -0.33 | -3.0 | 0.11 | Liver |
| *UNG* | 0.059 | -0.30 | -1.9 | 0.15 | Esophagus - Gastroesophageal Junction |
| *UNG* | 0.070 | -0.28 | -1.8 | 0.15 | Pituitary |
| *UNG* | 0.030 | -0.24 | -2.2 | 0.11 | Pancreas |
| *UNG* | 0.021 | -0.20 | -2.3 | 0.09 | Skin - Not Sun Exposed (Suprapubic) |
| *UNG* | 0.005 | -0.17 | -2.8 | 0.06 | Skin - Sun Exposed (Lower leg) |
| *UNG* | 0.016 | -0.15 | -2.4 | 0.06 | Lung |
| *UNG* | 0.054 | -0.16 | -1.9 | 0.08 | Artery - Coronary |
| *UNG* | 0.035 | -0.16 | -2.1 | 0.08 | Adipose - Visceral (Omentum) |
| *UNG* | 0.360 | -0.13 | -0.9 | 0.14 | Colon - Sigmoid |
| *UNG* | 0.044 | -0.09 | -2.0 | 0.04 | Muscle - Skeletal |
| *UNG* | 0.023 | -0.17 | -2.3 | 0.07 | Breast - Mammary Tissue |
| *UNG* | 0.590 | -0.10 | -0.5 | 0.19 | Uterus |
| *UNG* | 0.220 | -0.15 | -1.2 | 0.12 | Vagina |
| *UNG* | 0.140 | -0.16 | -1.5 | 0.11 | Ovary |
| *UNG* | 2.5e-7 | -0.19 | -5.3 | 0.04 | Whole Blood |
| *^a^*Nominal eQTL p-values were generated for each SNP-gene pair using a two-tailed t test, testing the alternative hypothesis that β (slope of the linear regression model) deviates from the null hypothesis of β=0. | | | | | |
|  |  |  |  |  |  |
